# Match Physical and Physiological Response of Amateur Soccer Referees: A Comparison between Halves and Match Periods

**DOI:** 10.3390/ijerph19031306

**Published:** 2022-01-24

**Authors:** Eñaut Ozaeta, Uxue Fernández-Lasa, Inmaculada Martínez-Aldama, Ruth Cayero, Daniel Castillo

**Affiliations:** 1 Faculty of Education and Sport, University of the Basque Country, UPV/EHU, 01007 Vitoria-Gasteiz, Spain; eozaeta001@gmail.com; 2 Society, Sports and Physical Exercise Research Group (GIKAFIT), Physical Education and Sport Department, Faculty of Physical Activity and Sports Science, University of the Basque Country, UPV/EHU, 01007 Vitoria-Gasteiz, Spain; uxue.fernandez@ehu.eus; 3 Physical Education and Sport Department, Faculty of Education and Sport, University of the Basque Country, UPV/EHU, 01007 Vitoria-Gasteiz, Spain; inmaculada.martinezdealdama@ehu.eus (I.M.-A.); ruth.cayero@ehu.eus (R.C.); 4 Faculty of Education, University of Valladolid, 42004 Soria, Spain

**Keywords:** field referees, assistant referees, match periods, external load, heart rate

## Abstract

The aim of this paper was to examine the differences in the external and internal load in amateur match officials between the 1st and 2nd half and among different 15 min periods. Twenty-three field referees (FRs) and 46 assistant referees (ARs) from the Spanish División de Honor participated in this study. Match external and internal loads were monitored showing that FRs recorded a lower Power_mean_, Speed_mean_, Cadence_mean_ and Stiffness_mean_ (*p* < 0.05; d = 0.52 to 0.57) during the 2nd half and they also recorded a lower HR_mean_, and HR_peak_, and spent less time in zone 5 (*p* < 0.05; d = 0.50 to 0.62). The FRs’ match load decreased during the match but they performed higher Power_mean_ and covered more distance in the last 15 min of the match (*p* < 0.01; d = 0.87 to 4.28). The ARs external load did not show significant variations between halves, but ARs recorded a lower HR_mean_ and spent less time in zone 5 (*p* < 0.01; d = 0.41 to 0.63), and the highest values of Power_mean_, Speed_mean_, Cadence_mean_ and Vertical oscillation_mean_ during the first 15 min of the match (*p* < 0.05; d = 0.45 to 0.75). The highest values of HR_mean_ and distance covered were in the 0–15 min period. Results suggest that match load decreases as the match progresses because of the neuromuscular fatigue but increases in the last 15 min.

## 1. Introduction

Field referees (FRs) and assistant referees (ARs) are responsible for controlling and supervising football matches. To be able to carry out their role, from a physical and physiological point of view, referees perform several intermittent actions throughout the match, spending time standing, walking, running at different intensities and also moving in non-orthodox modes [1,2]. Both FRs and ARs must have an adequate physical condition that allows them, on the one hand, to be correctly positioned during the match and, on the other hand, to follow the match pace, in order to make correct decisions [3]. Due to the importance of the physical and physiological dimension of refereeing, the FRs’ and ARs’ physical condition [4,5,6] and match load [7,8,9] have been the subject of study in recent years. With regard to match load, several studies have gone into greater depth in the analysis of external and internal match load [10,11,12].

Whereas the FRs cover the entire field of play, the ARs are limited to the touchline, which is why differences have been described in external and internal match loads [13,14]. With regard to the external load, while FRs cover a total distance of between 9 km and 12 km per match [1,8,15], ARs cover a total distance of ≈6 km per match [13,15], which is approximately 33% to 50% less than the FRs’ total distance. It has also been found that the distance covered at different intensities is higher in FRs in comparison to ARs [13,14]. FRs covered more distance at high intensity (>13 km·h^−1^) [16] than ARs. Similarly, FRs covered 798.32 ± 310.19 m at high speed (>18 km·h^−1^) which is 66% less than the distance covered by ARs at high speed 273.74 ± 132.11 m [10]. Moreover, internal match load has also been studied demonstrating differences between FRs and ARs. For example, mean heart rate (HR_mean_) in FRs is over 85%of their maximal heart rate (HR_max_) [17,18,19] while in ARs the HR_mean_ is 77% of their HR_max_ [20]. Similarly, the HR_mean_ in FRs is 152.87 ± 11.44 bpm and in ARs is 132.73 ± 13.63 bpm, as described by Castillo et al. [6]. Regarding match load, FRs showed higher Edwards Training Impulse (TRIMP) and Stagno’s TRIMP values than ARs [9,21]. Current knowledge seems to show that external and internal loads are higher for FRs than ARs.

While many studies have focused on analyzing external and internal load in FRs and ARs during the entire match, fewer studies have focused on analyzing whether there are differences between the 1st and 2nd half [22,23]. It has been observed that FRs covered less distance in the 2nd half than in the 1st half [24,25] and they also performed fewer accelerations, distance covered in accelerating [23] and distance at high speed [22]. When internal load is observed, FRs HR_mean_ and peak heart rate (HR_peak_) are lower in the 2nd half compared to the 1st half [14,26]. Similarly, ARs showed less distance covered in the 2nd half compared to the 1st half [25] and less distance covered at high speed [22]. Besides analyzing the differences in the external and internal match load between the 1st and 2nd half, some studies have investigated the evolution of the external and internal load in different shorter periods during the match (i.e., 15 min) [13,15,27]. Knowing the evolution of the load in shorter periods can provide relevant information on the physical and physiological requirements at different times of the match. Previous studies in both FRs and ARs have observed that both external and internal loads, measured by different variables, appear to be higher in the initial periods of the match and that there is a decline in the load as the match progresses [13,15,18,27]. Similarly, it has been found that some external and internal load variables are lower in the final periods of both the 1st and 2nd half [15,18,28]. However, most of the published studies have been carried out with high level match officials [13,17,28] and no studies have analyzed the evolution of the load at different moments of the match (i.e.,15 min periods) in amateur referees. Knowing officials’ match load evolution can be especially relevant because it provides more detailed information on the distribution of the physical and physiological load, and it would be interesting to study the evolution of the load in amateur referees.

Therefore, the objectives of this study were to describe the differences in external and internal load in amateur match officials (FRs and ARs) between the 1st and 2nd half, and to analyze the differences among 15 min periods of the match (0–15 min, 15–30 min, 30–45 min, 45–60 min, 60–75 min and 75–90 min).

## 2. Materials and Methods

### 2.1. Participants

Sixty-nine Spanish match referees, who officiated soccer matches in the Spanish “Division de Honor” during the 2019–2020 competitive season, participated in the present study. Twenty-three of them were FRs (age: 25.65 ± 3.30 years; height: 173.4 ± 3.81 cm; body mass: 64.86 ± 5.82 kg; body mass index (BMI): 21.56 ± 1.67 kg·m^−2)^ and 46 were ARs (age: 23.11 ± 4.15 years; height: 178.15 ± 4.30 cm; body mass: 72.84 ± 6.67 kg; BMI: 22.94 ± 1.87 kg·m^−2^). They had officiated soccer matches at this competitive level for at least three years. All the participants trained at least twice a week and were involved in refereeing in this category on average twice per month. This investigation was performed in accordance with the Declaration of Helsinki (2013) and was approved by the Ethics Committee (CEISH) of The University of the Basque Country (UPV/EHU) (Code: M10/2018/289).

### 2.2. Procedures

To carry out this study 23 official matches were recorded during the in-season period (i.e., from December to February). Throughout all the matches, external match load (distance, power, speed, cadence, vertical oscillation, ground contact time and stiffness) and internal match load (heart rate, HR) were recorded in both FRs’ and ARs’ groups. A comparative design was used to examine the differences between the 1st and 2nd half and among periods of 15 min both in external and internal match load. Data was collected during 23 official in-season matches (i.e., from December to February). All matches were played between 11 a.m. and 5 p.m. Before the beginning of the match all the officials performed a 10 min warm-up < consisting of running, stretching, short sprints and progressive sprints. The 15 min half-time data were excluded from the external and internal match load analysis.

### 2.3. Measures

External match loads. External loads were monitored using a Stryd Power Meter (Stryd, Inc., Boulder, CO, USA) placed on the laces of the right soccer boot with a plastic clip [29]. Stryd Power Meter was used to carry out this study because its repeatability has been demonstrated (SEM ≤ 12.5 W, CV ≥ 4.3 %, ICC ≤ 0.989) [30]. Offline data collection was activated in accordance with the manufacturer’s recommendations. The external match load data were collected as measures of total distance covered (km), average power (Power_mean_, W), average speed (Speed_mean_, km·h^−1^), average cadence (Cadence_mean_, steps·min^−1^), average vertical oscillation (Vertical oscillation_mean_, cm), average ground contact time (GCT_mean_, m·s^−1^) and average stiffness (Stiffness_mean_, KN·m^−1^).

Internal match loads. Match officials’ HR was recorded during the whole match with a Polar Team 2 device (Polar Team System^™^, Kempele, Finland) according to the method previously used with football match officials by Castillo et al. [6]. The TRIMP based on Edwards (1993) was calculated by the sum of the values obtained multiplying the time spent in five arbitrary HR zones (from zone 1 to zone 5) by the value of each zone [9,31]. The zones were calculated as a percentage of peak HR (HR_peak_) obtained during the whole match [7]. The TRIMP value is represented in arbitrary units (AU).

### 2.4. Statistical Analysis

Results are presented as mean ± standard deviation. Normal distribution and homogeneity of variances were tested using the Kolmogorov–Smirnov and Levene tests. Student’s *t*-test for paired samples was performed in order to evaluate mean differences between the 1st and 2nd half in FRs‘ and ARs’ external and internal match loads. Repeated measures of ANOVA and Bonferroni’s post hoc were used to analyze mean differences among the periods of 15 min (0–15, 15–30, 30–45, 45–60, 60–75 and 75–90 min) in FRs’ and ARs’ external and internal match loads. Practical significance was assessed by Cohen’s d [32], with values of above 0.8, between 0.8 and 0.5, between 0.5 and 0.2, and lower than 0.2 being considered large, moderate, small, and trivial, respectively. We performed all statistical power calculations via G*Power 3.1.9.2 (Universität Kiel, Kiel, Germany) and supported this sample size for comparisons between two dependent means using power = 0.80, α = 0.05, and effect size = 0.50. Considering the ANOVA test for repeated measures, the total sample size (n = 23), an effect size f of 0.25, an alpha error of 0.05 and a 0.5 of correlation among repeated measures, we obtained a statistical power of 90%. The data analysis was carried out using JASP (JASP Team (2021), JASP version 0.16, Amsterdam, The Netherlands). Statistical significance was set at *p* < 0.05.

## 3. Results

The differences recorded by FRs between the 1st and 2nd half in external and internal loads during the official matches are shown in Table 1. When external loads are observed, FRs recorded lower Power_mean_ (*p* < 0.05; d = 0.54, moderate), Speed_mean_ (*p* < 0.05; d = 0.54, moderate), Cadence_mean_ (*p* < 0.05; d = 0.52, moderate) and Stiffness_mean_ (*p* < 0.05; d = 0.57, moderate) in the 2nd half than in the 1st half. No significant differences (*p* > 0.05) were found in distance, vertical oscillation or ground contact time between both halves. FRs spent more time in zone 2, zone 3 and zone 4 (*p* < 0.05; d = 0.45 to 0.69, moderate) in the 2nd half compared to the 1st half, and spent less time in zone 5 (*p* < 0.01; d = 0.62, moderate) during the 2nd half. In addition, FRs recorded lower values in HR_mean_ (*p* < 0.05; d = 0.56, moderate) and HR_peak_ (*p* < 0.05; d = 0.50, small) in the 2nd half compared to the values obtained in the 1st half.

The differences recorded by the FRs among 15 min periods in external and internal loads during the official matches are shown in Table 2. FRs covered more distance in the last 15 min period (75–90 min) than in any other (*p* < 0.01; d = 0.87 to 1.45, large). Moreover, in the 75–90 min period higher Power_mean_ values were recorded than in the 0–15 min (*p* < 0.01; d = 4.09, large), 30–45 min (*p* < 0.01; d = 4.14, large) and 60–75 min (*p* < 0.01; d = 4.28, large) periods. Higher values of Power_mean_ were also recorded in the 15–30 min and 45–60 min periods compared to 0–15 min, 30–45 min and 60–75 min (*p* < 0.01; d = 4.34 to 4.56, large). Speed_mean_ was higher in the 0–15 min period than in the 30–45 min, 60–75 min and 75–90 min periods (*p* <.01; d = 0.87 to 0.89, large). In addition, FRs recorded higher Cadence_mean_ in the 0–15 min period than in the 30–45 min, 60–75 min and 75–90 min periods (*p* < 0.01; d = 0.77 to 0.88, moderate to large).

Regarding the internal load, a lower HR_mean_ was recorded in the 45–60 min period than in the 15–30 min period (*p* < 0.05; d = 0.70, moderate). Furthermore, lower HR_peak_ values were recorded in the 45–60 min than the 0–15 min (*p* < 0.01; d = 0.79, moderate) and the 15–30 min (*p* < 0.01; d = 0.68, moderate) periods. In addition, lower HR_peak_ values were recorded in the 60–75 min than the 0–15 min (*p* < 0.05; d = 0.70, moderate) period. FRs spent more time in zone 3 and zone 4 in the 75–90 min period than in the 15 min periods of the 1st half (*p* < 0.01; d = 0.64 to 0.96, moderate to large). FRs also spent more time in zone 5 in the 15–30 min period than in the 45–60 min (*p* < 0.05; d = 0.68, moderate) and the 60–75 min (*p* < 0.05; d = 0.68, moderate) periods. Finally, FRs recorded higher TRIMP values in the 75–90 min period than in the rest of the 15 min periods of the match (*p* < 0.01; d =1.20 to 1.97, large). FRs also recorded higher TRIMP in the 30–45 min than in the 45–60 min and 60–75 min periods (*p* < 0.05; d = 0.72 to 0.78, moderate).

The differences recorded by ARs between the 1st half and 2nd half in external and internal loads during official matches are shown in Table 3. In ARs, no significant decrease of external load was observed in the 2nd half except in Vertical Oscillation_mean_ (*p* < 0.01; d = 0.42, moderate)_._ However, higher values of Cadence_peak_ were recorded in the 2nd half (*p* < 0.05; d = 0.35, small). Despite no significant differences being found in most of the analyzed variables of external load between the 1st and 2nd half, when internal load is studied, ARs showed lower HR_mean_ values in the 2nd half (*p* < 0.01; d = 0.63, moderate) and they also spent less time in zone 5 (*p* < 0.01; d = 0.41, small). On the other hand, ARs spent more time in the 2nd half than in the 1st half in zones 1 and 2 (*p* < 0.01; d = 0.42 to 0.48, small).

The differences recorded by ARs among 15 min periods in external and internal loads during the official matches are shown in Table 4. The periods in which more distance was covered were 30–45 min (*p* < 0.01; d = 0.59 to 0.62, moderate) and 75–90 min (*p* < 0.01; d = 0.51 to 0.97, moderate to large). More distance was covered also in 0–15 min compared to 45–60 min (*p* < 0.05; d = 0.45, small). Moreover, in the 0–15 min period ARs recorded higher Power_mean_, than in the 30–45 min, 60–75 min and 75–90 min periods (*p* < 0.01; d = 0.53 to 0.58, moderate); higher Speed_mean_ than in 30–45 min and 60–75 min (*p* < 0.05; d = 0.50 to 0.55, small to moderate), higher Cadence_mean_ than in 15–30 min, 30–45 min, 60–75 min and 75–90 min periods (*p* < 0.01; d = 0.57 to 0.75, moderate) and higher Vertical oscillation_mean_ than in the 60–75 min and 75–90 min periods (*p* < 0.05; d = 0.44 to 0.60, small to moderate). In addition, the 45–60 min period also showed higher Cadence_mean_ values than the 30–45 min, 60–75 min and 75–90 min periods (*p* < 0.05; d = 0.44 to 0.50, small). Finally, in the 45–60 min period lower GCT_mean_ values were shown than in the 30–45 min and 75–90 min periods (*p* < 0.01; d = 0.52 to 0.62, moderate), and lower GCT_peak_ values than in the 0–15 min, 30–45 min and 75–90 min periods (*p* < 0.05; d = 0.45 to 0.47, small).

When internal load is observed, ARs recorded lower HR_mean_ values in the 45–60 min, 60–75 min and 75–90 min periods than in the 0–15 min period (*p* < 0.01; d = 0.63 to 0.83, moderate to large). HR_mean_ lower values were also recorded in the 45–60 min and 60–75 min periods than in the 15–30 min period (*p* < 0.05; d = 0.45 to 0.46, small). In addition, the 60–75 min period recorded a lower HR_peak_ than the 0–15 min period (*p* < 0.05; d = 0.44, small). ARs spent more time in zones 1 and 2, as the match progressed (*p* < 0.05; d = 0.44 to 0.81, small to large). Moreover, ARs were in zone 3 more times in the 75–90 min period than in the previous 15 min periods (*p* < 0.01; d = 0.46 to 0.67, small to moderate) and also spent more time in zone 4 during the 75–90 min period than in the 45–60 min and 60–75 min periods (*p* < 0.05; d = 0.45 to 0.46, small). In contrast to zones 1 and 2, ARs spent less time in zone 5 as the match went on, recording more time in zone 5 during the 0–15 min period than in the 30–45 min, 45–60 min, 60–75 min and 75–90 min periods (*p* < 0.05; d = 0.47 to 0.81, small to large). They also spent less time in zone 5 during the 60–75 min period than in the 15–30 min period (*p* < 0.01; d = 0.52, moderate).

## 4. Discussion

There are studies that have analyzed the differences in the external and internal load between the 1st and 2nd half or between shorter periods in FRs and ARs during match play [13,14,22,23]. However, this is the first study to analyze the differences in external and internal load among 15 min periods in amateur match officials. Considering that there are thousands of amateur official matches officiated by amateur referees, a more exhaustive knowledge of the evolution of the external and internal load during match play could be interesting in order to adjust the training sessions for optimizing match officials’ performance.

The results of this study showed that FRs recorded lower Power_mean_, Speed_mean_, Cadence_mean_ and Stiffness_mean_ during the 2nd half in comparison to the 1st half, but no differences were found in Vertical oscillation_mean_ and GCT_mean._ These results are in concordance with other studies which found a lower external load during the 2nd half in international match officials [23,25]. Similarly, although FRs spent more time in zones 2, 3 and 4 during the 2nd half compared to the 1st half, they recorded a significantly lower HR_mean_, HR_peak_ and time spent in zone 5. Again, these results coincide with previous literature in national and international match officials [14,26]. One of the main contributions of the present study is that, despite a decrease being found in the external load average variables, no differences were found between any external load peak variables. These results show that FRs are able to perform similar peak values during the 2nd half compared to the 1st half but are not capable of maintaining mean external load. This was corroborated by Costa et al. [33] reporting that FRs’ mean speed decreases during the 2nd half but they can maintain a similar maximum speed during match play. In future studies it would be necessary to analyze whether the decrease in the external and internal load during the 2nd half in FRs is due to neuromuscular and/or cardiovascular fatigue or to other factors derived from the match.

To achieve a more in-depth analysis of external and internal load variations in the 1st and 2nd half, a study of shorter periods can provide more information about what happens during match play [13,15,27,34]. Our results showed highest Speed_mean_ and Cadence_mean_ at the beginning of the match (0–15 min) that were significantly higher than in the 30–45 min, 60–75 min and 75–90 min time-periods. These results are in concordance with previous studies in which the first 15 min periods revealed the greatest external loads during international matches [13,15]. On the other hand, FRs covered more total distance during the last 15 min of the match (75–90 min) in comparison to other 15 min periods of match play. In addition, higher Power_mean_ was recorded during the 15–30 min, 45–60 min and 75–90 min periods with respect to other 15 min periods. Nevertheless, no significant differences were found in Vertical oscillation_mean_, GCT_mean_ and Stiffness_mean_ during 15 min time periods across match play. Regarding internal load, although the variables decrease as the match progresses, our results also showed that the time in zones 3, 4 and 5 was longer than in previous 15 min periods during the 2nd half. These findings could be explained by higher match intensity caused by the increase in the number of missed passes [35,36] which could translate into more transitions involving more disordered match play, so FRs have to be continuously close to play in order to be more successful making key decisions [37].

Since the ARs perform specific movement patterns during match play, it is interesting to know the variations in the external and internal load throughout official matches. Our results showed that there were no significant differences in the variation of external loads between halves in ARs, with the exception of Vertical oscillation_mean_ and Cadence_peak_ decreasing and increasing during the 2nd half, respectively. Related to variation in internal load across matches, a decrease was observed in the HR_mean_ and time spent in zone 5 during the 2nd half. Otherwise, time spent in zones 1 and 2 increased during the 2nd half in ARs which is concordant with the study of Castillo et al. [6] with national ARs. Overall, the lack of differences in external loads between halves could be due to the different roles of ARs during match play with respect to FRs, who are exposed to greater external loads because they have to cover more ground [9,27]. On the other hand, according to the differences reported in internal load, it could be that ARs spent less time moving backwards and sideways [27] which create more metabolic demand [38] maintaining external load similar to the 1st half but increasing the internal load during the 2nd half.

To understand the external and internal loads as the match progresses in ARs, a 15 min time-period analysis is necessary. Our results showed that ARs recorded the highest values of Power_mean_, Speed_mean_, Cadence_mean_ and Vertical oscillation_mean_ during 0–15 min period. However, it was observed that ARs covered more total distance during the last 15 min of both halves (i.e., the 30–45 min and 75–90 min periods) than other 15 min periods. Similarly, to the external load, when internal load is observed, the higher values of HR_mean_ and time spent in zone 5 were recorded in 0–15 min and were significantly higher than in all 15 min periods of the 2nd half (45–60 min, 60–75 min and 75–90 min) (*p* < 0.05; d = 0.45 to 0.83, small to large). As occurs with FRs, it seems that the last minutes of the match imply that soccer players miss more passes [35] which could cause more transitions in play in which ARs have to cover greater distances.

In the present study it was not possible to analyze the reasons for the variation in the internal or external load in FRs and ARs in the different periods of the match. For this reason, it would be interesting to analyze in future studies what these reasons might be and whether they might be due, among other things, to motivation, the result of the match, fatigue or other factors.

## 5. Conclusions

The results of this study show that both FRs’ and ARs’ match load decreases during the match except for the last 15 min period, in which FRs and ARs covered more distance and FRs also produced higher Power_mean_. Results suggest that match load decreases as the match progresses probably caused by neuromuscular fatigue. However, in the last 15 min some match load variables increased in both FRs’ and ARs’ groups due to a higher number of attacks and transitions increasing their match load.

## Figures and Tables

**Table 1:** Differences in external and internal match loads between 1st half and 2nd half in FRs.

	1st Half	2nd Half	*p* < Value	Cohen’s d
*External loads*				
Distance (km)	4.30 ± 0.48	4.35 ± 0.45	0.52	−0.14
Power_mean_ (W)	122.80 ± 11.81	118.67 ± 12.46	0.02 *	0.54
Power_peak_ (W)	402.5 ± 54.94	402.98 ± 75.98	0.97	−0.01
Speed_mean_ (km·h^−1^)	7.22 ± 0.63	7.01 ± 0.69	0.02 *	0.54
Speed_peak_ (km·h^−1^)	23.32 ± 2.06	23.24 ± 1.96	0.86	0.04
Cadence_mean_ (steps·min^−1^)	63.32 ± 2.05	62.33 ± 1.95	0.02 *	0.52
Cadence_peak_ (steps·min^−1^)	119.63 ± 16.69	122.28 ± 19.38	0.60	−0.11
Vertical oscillation_mean_ (cm)	8.04 ± 0.56	7.96 ± 0.54	0.25	0.25
Vertical oscillation_peak_ (cm)	38.14 ± 7.79	37.39 ± 11.22	0.77	0.06
GCT_mean_ (m·s^−1^)	538.10 ± 68.84	544.35 ± 58.47	0.53	
GCT_peak_ (m·s^−1^)	1879.09 ± 241.31	1937.30 ± 195.84	0.41	−0.17
Stiffness_mean_ (KN·m^−1^)	9.40 ± 0.66	9.16 ± 0.53	0.01 *	0.57
Stiffness_peak_ (KN·m^−1^)	33.02 ± 8.71	31.80 ± 6.85	0.54	0.13
*Internal loads*				
HR_mean_ (bpm)	160.48 ± 12.75	157.01 ± 12.02	0.01 *	0.56
HR_peak_ (bpm)	189.26 ± 13.30	183.00 ± 11.05	0.02 *	0.50
Zone 1 (min)	0.95 ± 2.28	0.41 ± 1.77	0.30	0.22
Zone 2 (min)	1.50 ± 3.51	3.01 ± 6.57	0.04 *	−0.45
Zone 3 (min)	9.23 ± 7.29	12.50 ± 9.19	0.00 **	−0.69
Zone 4 (min)	19.50 ± 4.54	22.18 ± 8.09	0.04 *	−0.46
Zone 5 (min)	14.06 ± 8.04	10.30 ± 7.04	0.01 *	0.62
TRIMP (AU)	179.92 ± 21.60	184.19 ± 25.54	0.20	−0.27

Note. GCT = ground contact time; HR = heart rate; TRIMP = training impulse. * Significant differences between 1st half and 2nd half at *p* < 0.05; ** Significant differences between 1st half and 2nd half at *p* < 0.01.

**Table 2:** Differences in external and internal match loads between 15 min periods in FRs.

	0–15 min	15–30 min	30–45 min	45–60 min	60–75 min	75–90 min	Periods Pair Comparison (Cohen’s d)
*External loads*							
Distance (km)	1.46 ± 0.22	1.43 ± 0.12	1.41 ± 0.27	1.38 ± 0.18	1.32 ± 0.16	1.66 ± 0.24	0–15 and 75–90;
							−0.87 **
							15–30 and 75–90;
							−0.98 **
							30–45 and 75–90;
							−1.07 **
							45–60 and 75–90;
							−1.22 **
							60–75 and 75–90;
							−1.45 **
Power_mean_ (W)	127.08 ± 15.91	366.06 ± 69.24	124.00 ± 15.81	364.76 ± 60.68	116.71 ± 11.91	350.76 ± 50.36	0–15 and 15–30;
							−4.37 **
							0–15 and 45–60;
							−4.34 **
							0–15 and 75–90;
							−4.09 **
							15–30 and 30–45;
							4.42 **
							15–30 and 60–75;
							4.56 **
							30–45 and 45–60;
							−4.40 **
							30–45 and 75–90;
							−4.14 **
							45–60 and 60–75;
							4.53 **
							60–75 and 75–90;
							−4.28 **
Power_peak_ (W)	366.06 ± 69.24	364.76 ± 60.68	350.76 ± 50.36	357.46 ± 55.20	350.67 ± 83.50	366.50 ± 62.14	
Speed_mean_ (km·h^−1^)	7.43 ± 0.80	7.27 ± 0.73	6.92 ± 0.62	7.20 ± 0.73	6.93 ± 0.73	6.93 ± 0.82	0–15 and 30–45; 0.89 **
							0–15 and 60–75; 0.88 **
							0–15 and 75–90; 0.87 **
Speed_peak_ (km·h^−1^)	22.04 ± 2.78	21.86 ± 1.78	21.09 ± 2.57	21.82 ± 1.83	20.52 ± 2.76	21.51 ± 2.58	
Cadence_mean_ (steps·min^−1^)	64.46 ± 2.77	63.24 ± 2.50	61.99 ± 3.39	63.57 ± 2.42	61.97 ± 2.86	61.66 ± 2.35	0–15 and 30–45; 0.77 **
							0–15 and 60–75; 0.78 **
							0–15 and 75–90; 0.88 **
Cadence_peak_ (steps·min^−1^)	111.50 ± 14.44	107.11 ± 11.50	106.59 ± 15.08	108.15 ± 11.72	107.46 ± 16.18	113.50 ± 18.88	
Vertical oscillation_mean_ (cm)	8.13 ± 0.74	8.01 ± 0.51	7.94 ± 0.64	7.94 ± 0.50	8.08 ± 0.76	7.85 ± 0.64	
Vertical oscillation_peak_ (cm)	33.18 ± 8.94	28.87 ± 7.28	30.00 ± 9.07	30.72 ± 7.08	30.73 ± 8.47	33.02 ± 12.24	
GCT_mean_ (m·s^−1^)	517.98 ± 75.59	552.89 ± 67.08	544.20 ± 101.73	530.29 ± 77.86	551.47 ± 65.88	549.58 ± 64.44	
GCT_peak_ (m·s^−1^)	1761.65 ± 301.89	1676.87 ± 179.90	1671.04 ± 169.74	1718.96 ± 239.28	1725.91 ± 219.34	1826.22 ± 209.85	
Stiffness_mean_ (KN·m^−1^)	9.44 ± 0.87	9.40 ± 0.78	9.31 ± 0.60	9.18 ± 0.71	9.22 ± 0.65	9.08 ± 0.60	
Stiffness_peak_ (KN·m^−1^)	28.21 ± 7.87	26.35 ± 7.93	27.23 ± 7.89	26.93 ± 8.18	26.08 ± 5.12	26.43 ± 7.82	
*Internal loads*							
HR_mean_ (bpm)	160.22 ± 14.35	162.14 ± 12.30	159.14 ± 14.41	156.29 ± 12.75	157.00 ± 11.52	157.58 ± 13.05	15–30 and 45–60; 0.70 *
HR_peak_ (bpm)	185.83 ± 14.65	184.70 ± 12.84	180.65 ± 10.86	177.52 ± 12.84	178.44 ± 10.74	181.52 ± 10.38	0–15 and 45–60; 0.79 **
							0–15 and 60–75; 0.70 *
							15–30 and 45–60; 0.68 *
Zone 1 (min)	0.54 ± 1.74	0.10 ± 0.48	0.31 ± 1.42	0.19 ± 0.80	0.10 ± 0.42	0.12 ± 0.56	
Zone 2 (min)	0.42 ± 0.53	0.37 ± 0.91	0.71 ± 2.20	0.86 ± 1.84	0.91 ± 2.24	1.25 ± 2.76	
Zone 3 (min)	3.06 ± 2.41	2.62 ± 2.71	3.54 ± 2.97	4.01 ± 2.98	3.72 ± 3.09	4.77 ± 3.77	0–15 and 75–90;
							−0.76 **
							15–30 and 75–90;
							−0.96 **
Zone 4 (min)	6.10 ± 1.93	6.80 ± 1.98	6.60 ± 2.36	6.78 ± 2.49	7.12 ± 2.83	8.29 ± 3.67	0–15 and 75–90;
							−0.82 **
							30–45 and 75–90;
							−0.64 *
Zone 5 (min)	4.52 ± 2.74	4.85 ± 3.20	4.69 ± 3.25	2.94 ± 2.49	2.94 ± 2.06	4.42 ± 3.60	15–30 and 45–60; 0.68 *
							15–30 and 60–75; 0.68 *
TRIMP (AU)	57.55 ± 8.90	60.14 ± 6.86	62.23 ± 10.68	55.74 ± 8.39	56.25 ± 7.96	72.20 ± 11.49	0–15 and 75–90;
							−1.76 **
							15–30 and 75–90;
							−1.44 **
							30–45 and 45–60; 0.78 **
							30–45 and 60–75; 0.72 *
							30–45 and 75–90;
							−1.20 **
							45–60 and 75–90;
							−1.97 **
							60–75 and 75–90;
							−1.91 **

Note. GCT = ground contact time; HR = heart rate; TRIMP = training impulse. * Significant differences between periods at *p* < 0.05; ** Significant differences between period at *p* < 0.01.

**Table 3:** Differences in external and internal match loads between 1st half and 2nd half in ARs.

	1st Half	2nd Half	*p* < Value	Cohen’s d
*External loads*				
Distance (km)	1995 ± 0.46	1924 ± 0.56	0.46	0.11
Power_mean_ (W)	90.90 ± 10.45	88.77 ± 8.79	0.16	0.21
Power_peak_ (W)	322.35 ± 39.70	322.22 ± 46.64	0.99	0.00
Speed_mean_ (km·h^−1^)	5.84 ± 0.53	5.75 ± 0.50	0.25	0.17
Speed_peak_ (km·h^−1^)	20.86 ± 2.02	20.78 ± 2.42	0.84	0.03
Cadence_mean_ (steps·min^−1^)	56.40 ± 3.64	55.61 ± 3.50	0.06	0.29
Cadence_peak_ (steps·min^−1^)	127.85 ± 19.45	137.56 ± 23.48	0.02 *	−0.35
Vertical oscillation_mean_ (cm)	7.71 ± 0.77	7.41 ± 0.52	0.01 *	0.42
Vertical oscillation_peak_ (cm)	39.57 ± 8.56	37.18 ± 10.31	0.25	0.17
GCT_mean_ (m·s^−1^)	391.17 ± 204.35	366.05 ± 231.16	0.20	0.20
GCT_peak_ (m·s^−1^)	2015.74 ± 223.34	1924 ± 348.09	0.10	0.25
Stiffness_mean_ (KN·m^−1^)	8.69 ± 0.59	8.61 ± 0.67	0.19	0.20
Stiffness_peak_ (KN·m^−1^)	30.48 ± 11.20	32.16 ± 11.80	0.51	−0.10
*Internal loads*				
HR_mean_ (bpm)	136.86 ± 18.46	131.92 ± 18.26	0.00 **	0.63
HR_peak_ (bpm)	179.09 ± 23.08	177.50 ± 21.51	0.56	0.09
Zone 1 (min)	4.69 ± 6.04	6.88 ± 7.21	0.00 **	−0.48
Zone 2 (min)	10.22 ± 8.18	12.52 ± 8.38	0.01 *	−0.42
Zone 3 (min)	12.97 ± 6.36	14.14 ± 6.94	0.25	−0.17
Zone 4 (min)	11.82 ± 8.40	10.76 ± 9.01	0.21	0.19
Zone 5 (min)	4.68 ± 5.30	2.86 ± 3.41	0.01 *	0.41
TRIMP (AU)	134.77 ± 40.30	127.71 ± 43.16	0.16	0.21

Note. GCT = ground contact time; HR = heart rate; TRIMP = training impulse. * Significant differences between 1st half and 2nd half at *p* < 0.05; ** Significant differences between 1st half and 2nd half at *p* < 0.01.

**Table 4:** Differences in external and internal match loads between 15 min periods in ARs.

	0–15 min	15–30 min	30–45 min	45–60 min	60–75 min	75–90 min	Periods Pair Comparison (Cohen’s d)
*External loads*							
Distance (km)	0.67 ± 0.22	0.61 ± 0.19	0.71 ± 0.18	0.56 ± 0.25	0.57 ± 0.19	0.80 ± 0.22	0–15 and 45–60; 0.45 *
							0–15 and 75–90;
							−0.51 **
							15–30 and 75–90;
							−0.76 **
							30–45 and 45–60; 0.62 **
							30–45 and 60–75; 0.59 **
							45–60 and 75–90;
							−0.97 **
							60–75 and 75–90;
							−0.94 **
Power_mean_ (W)	95.49 ± 15.73	89.50 ± 10.85	87.88 ± 10.59	91.68 ± 13.33	87.20 ± 13.40	87.69 ± 10.34	0–15 and 30–45; 0.53 **
							0–15 and 60–75; 0.58 **
							0–15 and 75–90; 0.54 **
Power_peak_ (W)	295.26 ± 45.84	290.06 ± 47.47	276.52 ± 43.30	274.50 ± 57.70	269.37 ± 56.49	295.11 ± 47.96	
Speed_mean_ (km·h^−1^)	6.07 ± 0.78	5.77 ± 0.48	5.70 ± 0.63	5.85 ± 0.70	5.66 ± 0.73	5.75 ± 0.63	0–15 and 30–45; 0.50 *
							0–15 and 60–75; 0.55 **
Speed_peak_ (km·h^−1^)	19.38 ± 2.86	18.89 ± 2.52	18.37 ± 2.24	18.05 ± 3.26	18.08 ± 3.15	19.33 ± 2.41	
Cadence_mean_ (steps per min^−1^)	58.42 ± 4.45	55.80 ± 4.04	55.21 ± 4.27	57.29 ± 4.49	54.99 ± 4.16	55.26 ± 4.68	0–15 and 15–30; 0.57 **
							0–15 and 30–45; 0.70 **
							0–15 and 60–75; 0.75 **
							0–15 and 75–90; 0.69 **
							30–45 and 45–60;
							−0.46 *
							45–60 and 60–75; 0.50 *
							45–60 and 75–90; 0.44 *
Cadence_peak_ (steps per min^−1^)	115.28 ± 16.85	113.50 ± 18.47	111.96 ± 16.19	119.96 ± 22.90	113.44 ± 23.08	118.87 ± 22.74	
Vertical oscillation_mean_ (cm)	7.90 ± 0.97	7.61 ± 1.05	7.52 ± 0.96	7.52 ± 0.71	7.43 ± 0.81	7.26 ± 0.70	0–15 and 60–75; 0.44 *
							0–15 and 75–90; 0.60 **
Vertical oscillation_peak_ (cm)	34.36 ± 10.16	29.91 ± 8.47	31.61 ± 9.72	30.18 ± 11.01	30.37 ± 11.00	28.99 ± 7.27	
GCT_mean_ (m·s^−1^)	339.127 ± 109.76	353.310 ± 118.09	387.593 ± 117.70	294.224 ± 141.42	334.895 ± 119.22	372.590 ± 106.11	30–45 and 45–60; 0.62 **
							45–60 and 75–90;
							−0.52 **
GCT_peak_ (m·s^−1^)	1822.00 ± 232.90	1772.26 ± 252.74	1816.74 ± 260.16	1651.13 ± 319.11	1729.61 ± 319.35	1814.09 ± 368.58	0–15 and 45 –60; 0.47 *
							30–45 and 45–60; 0.46 *
							45–60 and 75–90;
							−0.45 *
Stiffness_mean_ (KN·m^−1^)	8.76 ± 0.75	8.60 ± 0.78	8.58 ± 0.74	8.54 ± 0.97	8.50 ± 0.84	8.73 ± 0.94	
Stiffness_peak_ (KN·m^−1^)	23.25 ± 10.32	21.19 ± 9.60	21.05 ± 9.76	22.30 ± 11.66	20.56 ± 9.17	24.87 ± 10.95	
*Internal loads*							
HR_mean_ (bpm)	139.76 ± 19.67	135.83 ± 18.22	135.44 ± 19.90	131.04 ± 20.71	130.97 ± 18.47	133.21 ± 18.11	0–15 and 45–60; 0.83 **
							0–15 and 60–75; 0.83 **
							0–15 and 75–90; 0.62 **
							15–30 and 45–60; 0.45 *
							15–30 and 60–75; 0.46 *
HR_peak_ (bpm)	173.91 ± 22.52	168.70 ± 18.64	168.87 ± 21.26	168.11 ± 21.30	166.02 ± 20.18	170.48 ± 21.63	0–15 and 60–75; 0.44 *
Zone 1 (min)	1.22 ± 1.82	1.72 ± 2.33	1.82 ± 2.52	2.21 ± 2.76	2.16 ± 2.42	2.36 ± 2.90	0–15 and 45–60;
							−0.48 *
							0–15 and 60–75;
							−0.46 *
							0–15 and 75–90;
							−0.56 **
Zone 2 (min)	3.00 ± 3.06	3.59 ± 2.82	3.83 ± 3.02	3.66 ± 2.52	4.05 ± 3.01	4.95 ± 3.55	0–15 and 60–75;
							−0.44 *
							0–15 and 75–90;
							−0.81 **
							15–30 and 75–90;
							−0.56 **
							30–45 and 75–90;
							−0.46 *
							45–60 and 75–90;
							−0.54 **
Zone 3 (min)	4.14 ± 2.62	4.11 ± 2.44	4.93 ± 2.51	4.23 ± 2.38	4.41 ± 2.51	5.83 ± 2.78	0–15 and 75–90;
							−0.66 **
							15–30 and 75–90;
							−0.67 **
							45–60 and 75–90;
							−0.62 **
							60–75 and 75–90;
							−0.56 **
Zone 4 (min)	4.17 ± 3.05	3.72 ± 2.78	4.00 ± 3.23	3.18 ± 2.82	3.20 ± 2.77	4.23 ± 3.76	45–60 and 75–90;
							−0.46 *
							60–75 and 75–90;
							−0.45 *
Zone 5 (min)	1.99 ± 2.38	1.52 ± 1.89	1.21 ± 1.57	0.95 ± 1.45	0.65 ± 0.97	1.05 ± 1.21	0–15 and 30–45; 0.47 *
							0–15 and 45–60; 0.62 **
							0–15 and 60–75; 0.81 **
							0–15 and 75–90; 0.57 **
							15–30 and 60–75; 0.52 **
TRIMP (AU)	46.29 ± 13.56	43.72 ± 13.20	46.32 ± 13.47	39.68 ± 14.34	39.56 ± 12.53	51.89 ± 14.99	0–15 and 45–60; 0.75 **
							0–15 and 60–75; 0.76 **
							0–15 and 75–90; −0.63 **
							15–30 and 45–60; 0.46 *
							15–30 and 60–75; 0.47 *
							15–30 and 75–90;
							−0.92 **
							30–45 and 45–60; 0.75 **
							30–45 and 60–75; 0.76 **
							30–45 and 75–90;
							−0.63 **
							45–60 and 75–90;
							−1.38 **
							60–75 and 75–90;
							−1.39 **

Note. GCT = ground contact time; HR = heart rate; TRIMP = training impulse; * Significant differences between periods at *p* < 0.05; ** Significant differences between periods at *p* < 0.01.

## Data Availability

Author’s property.
